# Evaluation of Biomarkers for Sacituzumab Govitecan in Metastatic Non–Small Cell Lung Cancer: Insights From the EVOKE-01 Study

**DOI:** 10.1158/2767-9764.CRC-26-0070

**Published:** 2026-08-06

**Authors:** Enriqueta Felip, Peiwen Kuo, Pilar Garrido, Christos Chouaid, Giannis Mountzios, Marcello Tiseo, Davey B. Daniel, Marianna Zavodovskaya, Shan Tang, Linda Su-Feher, Riddhi Patel, Joseph K. Park, Juliane M. Jürgensmeier, Yvonne Summers

**Affiliations:** 1 Vall d’Hebron University Hospital, Vall d’Hebron Institute of Oncology, Barcelona, Spain.; 2 Gilead Sciences, Inc., Foster City, California.; 3 Hospital Universitario Ramón y Cajal, Madrid, Spain.; 4 Service de Pneumologie, Centre Hospitalier Intercommunal de Créteil, Créteil, France.; 5 Henry Dunant Hospital Center, Athens, Greece.; 6 Department of Medicine and Surgery, University of Parma, Parma, Italy.; 7 Medical Oncology Unit, University Hospital of Parma, Parma, Italy.; 8 Tennessee Oncology, Nashville, Tennessee.; 9 The Christie NHS Foundation Trust, Manchester, United Kingdom.

## Abstract

**Purpose:**

This exploratory analysis assessed predictive biomarkers for sacituzumab govitecan (SG) in the open-label, phase III EVOKE-01 study of SG versus docetaxel in non–small cell lung cancer (NSCLC).

**Patients and Methods:**

Trophoblast cell-surface antigen (Trop) 2 membrane protein expression was analyzed using immunohistochemistry in the Trop-2 biomarker-evaluable population (BEP). Circulating tumor DNA (ctDNA) analyses evaluated baseline and reduction of ctDNA with treatment and identified oncogenic driver alterations (*KRAS*, *TP53*, *KEAP1*, and *STK11*) and other relevant mutations in the ctDNA BEP.

**Results:**

The median overall survival (OS) with SG versus docetaxel was 8.9 versus 9.8 months [hazard ratio (HR) = 0.96; 95% CI, 0.68–1.38] and 11.8 versus 10.7 months (HR = 0.89; 95% CI, 0.60–1.32) in subgroups with Trop-2 histologic scores greater than or equal to the median and less than the median, respectively. As expected, the median OS was longest in patients with undetectable baseline ctDNA. Reduction in ctDNA was observed after the first cycle with either treatment. *KRAS* driver alterations and potential immunotherapy resistance mutations (*TP53*, *KEAP1*, and *STK11*) were negative prognostic factors. Patients with *TP53* mutations derived more benefit from SG than docetaxel. SG activity was observed independent of DNA damage repair (DDR) mutations.

**Conclusions:**

Trop-2 expression did not identify patients benefiting more from SG than from docetaxel. ctDNA reduction supported SG as active therapy in NSCLC. Evaluation of clinically relevant biomarkers in NSCLC identified *KRAS*, *TP53*, *STK11*, and *KEAP1* mutations as negative prognostic factors in these second line–treated patients.

**Significance:**

The biomarker analyses of the phase III EVOKE-01 trial found that neither Trop-2 expression nor DDR alterations predicted benefit with SG versus docetaxel. Reduction in ctDNA in both treatment arms confirmed the activity of SG in this population. Further evaluation of predictive biomarkers for SG response in NSCLC is warranted.

## Introduction

Sacituzumab govitecan (SG) is a first-in-class antibody–drug conjugate (ADC) comprising an anti–trophoblast cell-surface antigen (Trop) 2 antibody coupled to the SN-38 payload via a hydrolyzable linker (1). Trop-2 is a transmembrane glycoprotein involved in regulating cancer growth and invasion and highly expressed in several cancer types, including non–small cell lung cancer (NSCLC); SN-38 is an active metabolite of the topoisomerase I (TOP1) inhibitor irinotecan (2–4). SG is currently approved for metastatic triple-negative breast cancer and metastatic hormone receptor–positive/human epidermal growth factor receptor 2–negative (HR+/HER2−) breast cancer (1).

EVOKE-01, a phase III open-label randomized study, compared SG and docetaxel in advanced or metastatic NSCLC that progressed on/after platinum-based chemotherapy and anti–programmed cell death protein (ligand) 1 [PD-(L)1] treatment. Although statistical significance was not met at final analysis, SG showed numerical improvement in the primary endpoint of overall survival (OS) compared with docetaxel [median, 11.1 vs. 9.8 months; hazard ratio (HR) = 0.84; 95% confidence interval (CI), 0.68–1.04], which was consistent across squamous and nonsquamous histologies (5). In addition, SG showed clinically significant OS benefit versus docetaxel in patients with stable disease (SD) or progressive disease (PD) after their last anti–PD-(L)1–containing regimen in both histologies [3.5-month median OS increase; HR, 0.75 (95% CI, 0.58–0.97); ref. 5].

Biomarkers are central to diagnosis, prognosis, and treatment decisions in patients with NSCLC. Data on potential predictive biomarkers for SG are limited, including Trop-2 expression and markers in the DNA damage repair (DDR) pathways, which may sensitize tumor cells to SN-38 (6, 7). A recent study showed that Trop-2 is highly expressed in NSCLC independent of histology, oncogenic driver alterations, or PD-L1 expression, but data exploring clinical outcomes of SG treatment with Trop-2 expression in NSCLC are restricted (4). Conflicting reports exist on Trop-2 as a biomarker, with several indicating that Trop-2 may be a negative prognostic factor in NSCLC (8–10). EVOKE-01 is the first study to explore Trop-2 as a potential predictive marker for SG in NSCLC. Furthermore, Trop-2 has been proposed as a marker of resistance to immune checkpoint inhibition in advanced NSCLC (11). TOP1 is also upregulated in NSCLC (12) and inhibited by multiple agents, including irinotecan (13), supporting further exploration of DDR mutations, as tumors harboring defects in DDR pathways may exhibit increased sensitivity to the DNA-damaging payload of SG.

Circulating tumor DNA (ctDNA) detected via tumor-specific methylation patterns is a surrogate marker of disease burden and a potential prognostic biomarker in NSCLC (14, 15), with early reduction after therapy initiation correlating with better clinical outcomes and persistent or increasing levels indicating poor response or impending relapse (14, 16–18). ctDNA analyses also assess tumor genomic profiles (14, 15). Oncogenic driver alterations are recognized biomarkers for selecting targeted therapies in NSCLC, and clinically relevant alterations are associated particularly with adenocarcinoma histology (19). The most common oncogenic alterations occur in *EGFR*, *ALK*, *RET*, *ROS1*, *BRAF*, *MET*, *KRAS*, *NTRK*, and *ERBB2* (20). In addition, mutations in *TP53*, *KEAP1*, and *STK11* have been linked to a lack of benefit with immunotherapy, especially when co-occurring (21–23). Given that oncogenic driver alterations define biologically distinct subgroups of NSCLC and influence therapeutic decisions in clinical practice, we explored whether these biomarkers could identify patients who may experience differential benefit from SG.

We performed a comprehensive exploratory biomarker analysis from the EVOKE-01 study to identify potential predictive biomarkers for SG and explore known biomarkers for NSCLC. The primary question was whether any of these markers, especially Trop-2, the target of SG, could identify patient subgroups deriving greater benefit from treatment with SG. This is the first report to evaluate Trop-2 and other potential biomarkers for SG efficacy in patients with NSCLC.

## Patients and Methods

### Study design and patients

The EVOKE-01 (ClinicalTrials.gov, NCT05089734) study design has been described previously (5). In brief, eligible adult patients had stage IV NSCLC that progressed after platinum-based chemotherapy and anti–PD-(L)1–containing therapy and an Eastern Cooperative Oncology Group (ECOG) performance status score of 0 or 1. Patients with known actionable genomic alterations (AGA) must have received treatment with at least 1 appropriate tyrosine kinase inhibitor. A total of 603 patients were randomly assigned (1:1) to receive either SG (*n* = 299) or docetaxel (*n* = 304) and stratified by histology (squamous vs. nonsquamous), best response to last anti–PD-(L)1–containing therapy [complete response (CR) or partial response (PR) vs. PD/SD], and previous treatment for AGAs (yes vs. no). SG was administered at 10 mg/kg by intravenous infusion on days 1 and 8 of a 21-day cycle and docetaxel at 75 mg/m^2^ by intravenous infusion on day 1 of a 21-day cycle. Treatment continued until disease progression, unacceptable toxicity, or other discontinuation criteria were met. The study was conducted in accordance with the International Council on Harmonisation Good Clinical Practice guidelines, the Declaration of Helsinki, and any applicable local health authority and institutional review board or independent ethics committee requirements. The study protocol and all amendments were approved by the institutional review board or independent ethics committee at each participating site. All patients provided written informed consent.

### Sample collection and analysis

Archival tumor tissue samples for Trop-2 expression analyses were obtained before study treatment initiation and were collected either as formalin-fixed, paraffin-embedded (FFPE) blocks or cut from FFPE blocks from consenting patients as optional samples. Blood samples were collected in Streck cell-free DNA (cfDNA) blood collection tubes before treatment initiation and at cycle (C) 2 day (D) 1 (C2D1, predose). Blood samples were processed into cfDNA-containing plasma (24, 25).

### Trop-2 analysis

Trop-2 biomarker analyses were conducted in the Trop-2 biomarker-evaluable population (BEP; *n* = 380; SG, *n* = 193; docetaxel, *n* = 187), which represented 63% of the intent-to-treat (ITT) population and was defined as the subset of patients in the ITT population who had Trop-2 biomarker data available. Trop-2 membrane protein expression was evaluated using the validated EPR20043 immunohistochemistry (IHC) assay (Roche Tissue Diagnostics).

Each tumor cell was evaluated for membrane Trop-2 staining intensity and was categorized as negative (0), weak (1+), moderate (2+), or strong (3+). Trop-2 intensity was summarized for tissue samples as the percentage of membrane-positive tumor cells at intensity 1+ (I1), 2+ (I2), and 3+ (I3). Histologic scores (H-score) of tumor samples were calculated using the formula: (1 × I1) + (2 × I2) + (3 × I3). The proportion of cells at moderate/strong (I2 + I3) and any (I1 + I2 + I3) intensity was also calculated for tumor samples.

Multiple Trop-2 expression scoring metrics and cutpoints were derived and explored for subgroup analyses. OS with SG versus docetaxel was analyzed by Trop-2 membrane expression as a continuous variable, using H-score, moderate/strong intensity, and any intensity. There was no evidence of an interaction between treatment and Trop-2 expression as a continuous variable for OS in this study. Interaction *P* values from unstratified Cox models (0.6081 for Trop-2 H-score, 0.7255 for moderate/strong intensity, and 0.6080 for any intensity) were weak, indicating no clear evidence for dependency of treatment effect on OS outcome by baseline Trop-2 expression. Therefore, subgroups based on moderate/strong and any intensity were defined using medians, tertiles, and 50% and 75% intensity scores. Subgroup analyses were performed on the primary endpoint of OS from the final analysis and the secondary efficacy endpoints of progression-free survival (PFS), objective response rate (ORR), disease control rate (DCR), and duration of response (DOR; ref. 5) with the data cutoff date of November 29, 2023.

### ctDNA analysis

ctDNA analyses were conducted in the ctDNA BEP, defined as the subset of 497 patients (SG, *n* = 245; docetaxel, *n* = 252) with ctDNA data available at baseline (82% of the ITT), of whom 477 patients (79% of the ITT) also had samples available at C2D1. The Guardant Infinity assay, a tumor-agnostic, combined epigenomic (methylation based) and genomic (>800 genes) platform, was used to quantify ctDNA fraction and profile gene alterations of interest, including single-nucleotide variations (SNV), copy-number variations, insertion and deletion mutations, and gene fusions. Molecular profiling was conducted on baseline ctDNA samples. Region-level ctDNA fractions were calculated as the ratio between the counts of differentially methylated regions and universally methylated regions for regions identified from testing a large cohort of nontumor and tumor samples by Guardant as part of the assay development. Sample-level ctDNA fractions were determined by averaging the top 100 region-level ctDNA fractions. Baseline ctDNA subgroups were defined based on the overall ctDNA BEP median, as ctDNA below median ctDNA fraction, ctDNA above median ctDNA fraction, and ctDNA not detected. ctDNA subgroup analyses were performed using clinical outcomes [OS, PFS, and best overall response (BOR)].

The sample-level percentage reduction in ctDNA was calculated as the percentage decrease from baseline to C2D1 (26). ctDNA reduction subgroups were classified as ctDNA reduction less than 50%, ctDNA reduction greater than or equal to 50%, and ctDNA low (ctDNA not detected at either baseline or C2D1).

Oncogenic driver alterations of interest were those identified in the literature as activating in NSCLC and included alterations of *EGFR* [exon 19 and 20 insertion–deletion (indel), L858R, L861Q, T790M, G719A, G724S, S768I, and C797S], *KRAS* (G12A, G12C, G12D, G12F, G12V, G13C, G13D, G13F, Q61H, Q61L, and Q61R), *ERBB2* (amplification, exon 20 insertions), *BRAF* (V600E), *MET* (exon 14 skipping), and *ALK*, *ROS1*, *RET*, and *NTRK1/2/3* (gene fusions). To explore this subpopulation further, ctDNA data were combined with mutational data collected from study sites as part of the protocol (with additional alterations in *MET* and *NTRK*) which increased the sample size. Additional information on oncogenic driver alterations from electronic case report forms (eCRF) were collected from study sites. Baseline ctDNA alterations were merged with eCRF records for some analyses. For ctDNA, any gene harboring an abovementioned alteration at baseline was included in the analysis. For eCRFs, genomic variants were retained if they had clinically relevant annotations (e.g., pathogenicity or specific driver mutations), or if the patient was flagged as previously receiving targeted therapy linked to the corresponding genomic alteration. *MET* alterations were only available from eCRFs. Subgroups based on the mutation status [wild-type (WT) vs. mutant] were analyzed for correlations with OS. Mutations in *TP53*, *KEAP1*, and *STK11* that may confer resistance to immunotherapy were evaluated for their ability to predict SG efficacy versus docetaxel.

Kyoto Encyclopedia of Genes and Genomes (KEGG; ref. 27) DDR gene panels were used to identify DDR gene mutations from ctDNA data. Of the 175 KEGG DDR genes, 80 were included in the Guardant Infinity panel. Additional mutational analyses were conducted using a homologous recombination repair (HRR) 24-gene list. Analyzed HRR genes included SNVs and indel mutations in *ATM*, *ATR*, *BAP1*, *BARD1*, *BRCA1/2*, *BRIP1*, *CDK12*, *CHEK1/2*, *FANCA*, *FANCL*, *HDAC2*, *MRE11*, *NBN*, *PALB2*, *RAD50*, *RAD51*, *RAD51B*, *RAD51C*, *RAD51D*, *RAD54L*, *XRCC2*, and *XRCC3*. OS was assessed based on DDR gene mutation status.

### Statistical analysis

OS, PFS, and DOR were summarized using Kaplan–Meier estimates. HRs for OS and PFS subgroups were estimated using Cox proportional-hazards regression models. The 95% CIs were determined by the Brookmeyer and Crowley method for OS, PFS, and DOR and by the Clopper–Pearson exact method for ORR and DCR. The Wilcoxon rank-sum test was used for comparisons of Trop-2 expression and ctDNA fractions. The Pearson *χ*^2^ test was used to determine the association between categorical variables. A *P* value of 0.05 was considered the threshold of statistical significance unless stated otherwise.

## Results

### Patient demographics and baseline characteristics

The Trop-2 BEP and ctDNA BEP were generally representative of the ITT population for demographic and baseline characteristics (Supplementary Table S1). Supplementary Table S2 shows the representativeness of the study. The median study follow-up periods were similar among the ITT population, Trop-2 BEP, and ctDNA BEP; the median OS with SG versus docetaxel was 11.1 versus 9.8 months (HR = 0.84; 95% CI, 0.68–1.04) in the ITT population, 10.3 versus 10.2 months (HR = 0.93; 95% CI, 0.72–1.21) in the Trop-2 BEP, and 12.2 versus 10.2 months (HR = 0.77; 95% CI, 0.61–0.98) in the ctDNA BEP (Supplementary Fig. S1A–S1C). Consistent with OS results, the median PFS with SG versus docetaxel was 4.1 versus 3.9 months (HR = 0.92; 95% CI, 0.77–1.11) in the ITT population and 3.6 versus 4 months (HR = 1; 95% CI, 0.79–1.26) in the Trop-2 BEP.

### Trop-2 analysis

**Figure 1.**
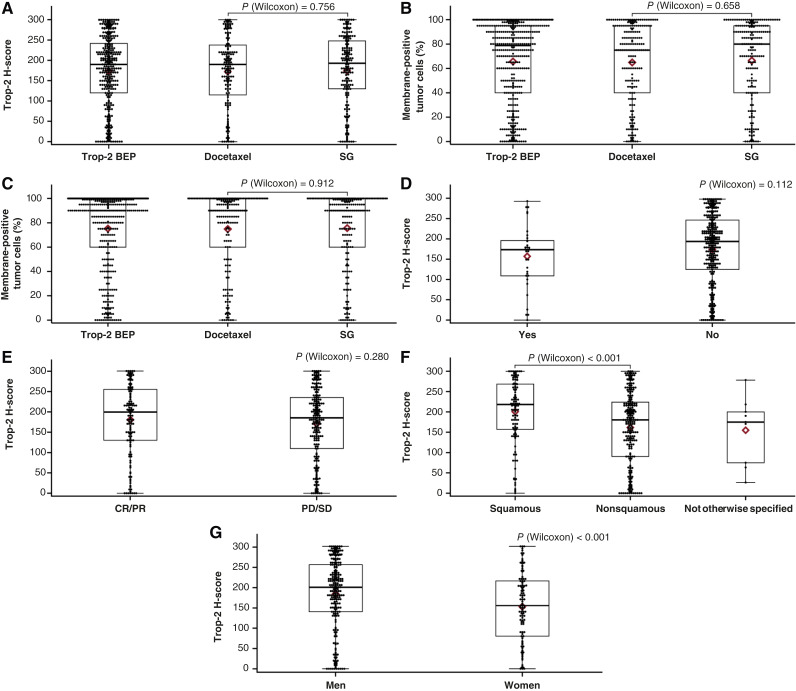
Trop-2 expression as (**A**) H-scores, (**B**) moderate/strong intensities, and (**C**) all intensities for the total Trop-2 BEP and by treatment arm, as well as H-scores in subgroups of patients based on (**D**) prior targeted therapy for AGA, (**E**) best response to last prior anti–PD-(L)1–containing therapy, (**F**) histology, and (**G**) sex. Distribution means are indicated by open diamonds. Bottom, middle, and top borders of boxes represent the first quartile (Q1), median, and third quartile (Q3), respectively. Whiskers represent the minimum and maximum values except for outliers that are greater than 1.5 times the interquartile range (Q3–Q1) from the median.

Trop-2 was highly expressed in tumor samples, with a median H-score of 190 (Fig. 1A) and median scores of 79% membrane-positive tumor cells for moderate/strong intensities and 90% for any intensity (Fig. 1B and C). Trop-2 H-score was similar in both treatment arms (Fig. 1A), as well as in subgroups based on prior targeted therapy for AGA, despite the small sample size for patients who received prior targeted therapy (Fig. 1D), and best response to last prior anti–PD-(L)1–containing therapy (Fig. 1E). Trop-2 H-scores were higher in squamous versus nonsquamous tumors (Fig. 1F) and in tumors from men versus women (Fig. 1G); the sex difference was unrelated to histology. Overall, Trop-2 expression results were consistent across scoring metrics.

**Figure 2.**
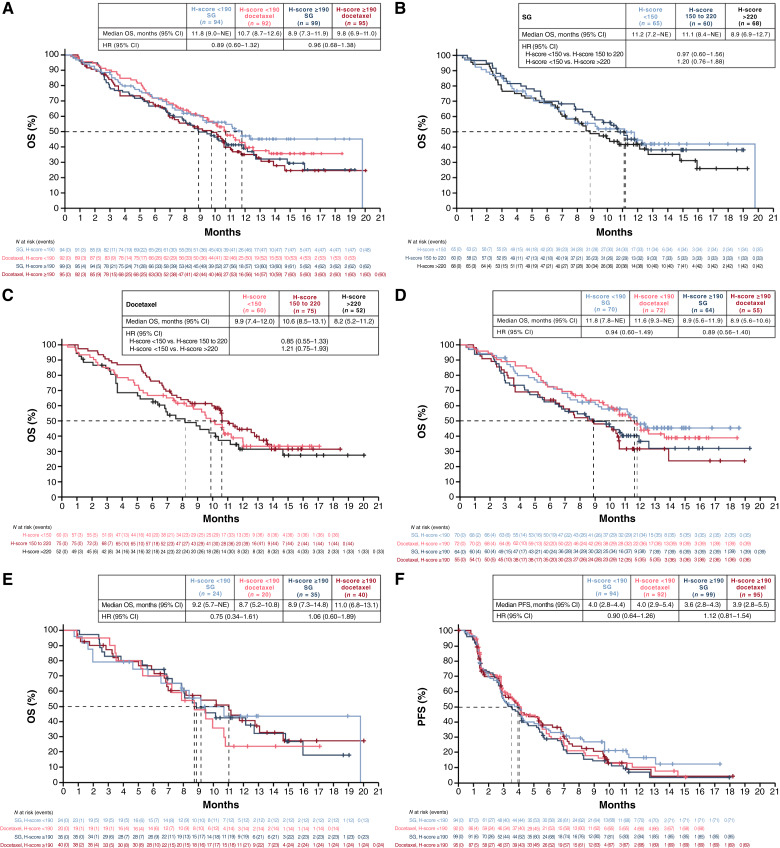
Kaplan–Meier plots in Trop-2 subgroups. OS by (**A**) median H-score subgroups across treatment arms, (**B**) H-score tertile subgroups with SG and (**C**) H-score tertile subgroups with docetaxel, (**D**) median H-score subgroups in nonsquamous histology subgroups across treatment arms, and (**E**) median H-score subgroups in squamous histology subgroups across treatment arms. **F,** PFS by median H-score subgroups across treatment arms. HR (95% CI) values are from an unstratified Cox regression analysis including treatment (SG or docetaxel) as the only predictor.

Higher Trop-2 expression did not predict OS improvement with SG versus docetaxel (Fig. 2). The median OS was 8.9 months with SG versus 9.8 months with docetaxel (HR = 0.96; 95% CI, 0.68–1.38) for patients with H-scores ≥ median and 11.8 months with SG versus 10.7 months with docetaxel (HR = 0.89; 95% CI, 0.60–1.32) for patients with H-scores < median (Fig. 2A). The median OS was shorter in patients with H-scores ≥ median in both treatment arms, indicating that Trop-2 is a negative prognostic factor. No statistical differences in OS were observed between subgroups based on Trop-2 H-score tertiles (tertile 1, <150; tertile 2, 150 to ≤220; tertile 3, >220) for SG (Fig. 2B) and docetaxel (Fig. 2C) treatment arms, respectively. Analyses using moderate/strong and any Trop-2 intensity subgroups were consistent with the above H-score results (Supplementary Fig. S2). In the nonsquamous subgroup, the median OS HR (95% CI) with SG versus docetaxel were 0.89 (0.56–1.40) and 0.94 (0.60–1.49) in the ≥ median Trop-2 H-score subgroups, respectively (Fig. 2D). Data in the squamous subgroup were consistent with the overall population; however, the sample size was small (Fig. 2E). Furthermore, no statistical differences in OS were observed for median Trop-2 H-score subgroups in patients with PD/SD (Supplementary Fig. S3A) and CR/PR (Supplementary Fig. S3B) as best response to last prior anti–PD-(L)1–containing therapy, respectively.

Analyses of PFS indicated no association with Trop-2 H-score subgroups: the median PFS was 4 months with SG and docetaxel (HR = 0.90; 95% CI, 0.64–1.26) for patients with H-scores ≥ median and 3.6 months with SG versus 3.9 months with docetaxel (HR = 1.12; 95% CI, 0.81–1.54) for patients with H-scores < median; these results were generally consistent with the results of the OS analysis (Fig. 2F). Furthermore, analyses using moderate/strong intensity and any Trop-2 intensity subgroups yielded results consistent with those of the H-score subgroups (Supplementary Fig. S4). No statistically significant differences in ORR and DCR were observed between arms when evaluating Trop-2 H-score subgroups (Supplementary Table S3).

### ctDNA analysis

**Figure 3.**
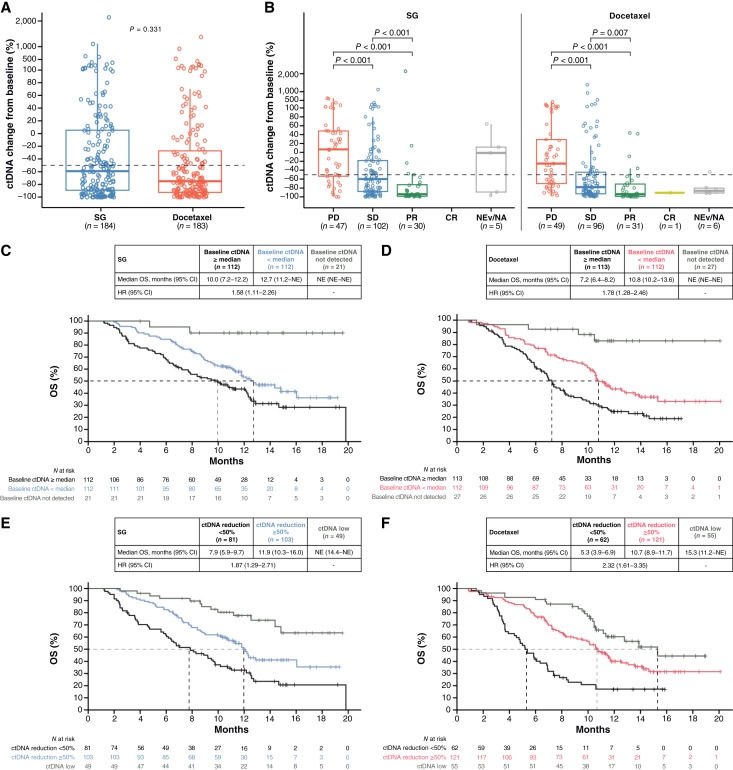
ctDNA percent change at C2D1 compared with baseline by (**A**) treatment arm and (**B**) BOR per Response Evaluation Criteria in Solid Tumors (RECIST) v1.1 and treatment arm. Kaplan–Meier plots for OS by baseline ctDNA subgroups with (**C**) SG and (**D**) docetaxel and by ctDNA reduction subgroups with (**E**) SG and (**F**) docetaxel. NA, not assessable; NEv, not evaluable.

Baseline ctDNA levels were similar for both treatment arms and were undetected in a similar number of patients receiving SG (*n* = 21) and docetaxel (*n* = 27). ctDNA levels at C2D1 were also similar between treatments (Supplementary Fig. S5A). ctDNA reduction was observed at C2D1 versus baseline, with a similar degree of reduction in the SG and docetaxel treatment arms (median reductions of 59% and 75%, respectively; Fig. 3A). Regardless of treatment, the median ctDNA reduction was greater among patients who experienced PR, followed by those with SD, and was the lowest in patients with PD as BOR (Fig. 3B).

Among baseline ctDNA subgroups, the median OS was longest [not estimable (NE)] in patients with undetectable baseline ctDNA, followed by patients with below median baseline ctDNA levels with both SG (Fig. 3C) and docetaxel (Fig. 3D). When assessing ctDNA reduction, the median OS was longest (NE with SG and 15.3 months with docetaxel) in patients with undetected ctDNA at either timepoint, followed by patients with 50% or more ctDNA reduction with SG (Fig. 3E) and docetaxel (Fig. 3F). PFS was consistent with OS data, with the longest median PFS (8.3 months with SG and 7.5 months with docetaxel) in patients with undetectable baseline ctDNA, followed by patients with below median baseline ctDNA levels (4.6 months with SG and 5.4 months with docetaxel; Supplementary Fig. S5B and S5C).

**Figure 4.**
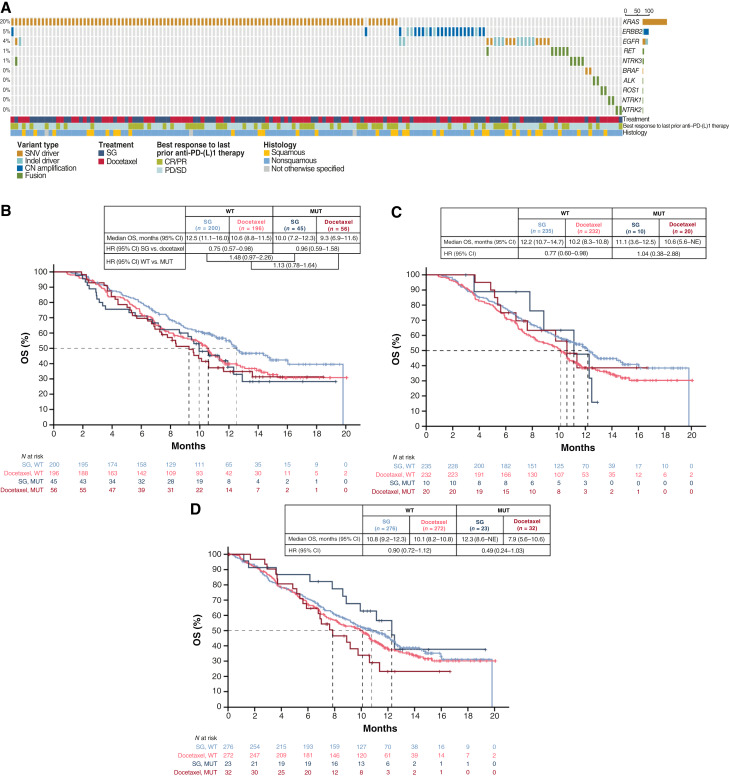
Oncogenic driver alterations. **A,** ctDNA oncogenic driver alteration landscape at baseline showing only cases harboring an alteration. Kaplan–Meier plots for OS by (**B**) *KRAS* mutation status and treatment arm, (**C**) *EGFR*, *ALK*, *ROS1*, and *RET* combined mutation status based on ctDNA only (ctDNA BEP), and (**D**) *EGFR*, *ALK*, *ROS1*, *RET*, *NTRK*, and *MET* combined mutation status based on ctDNA and eCRFs (ITT population). *KRAS* mutation status was defined as either WT or mutated, independent of other genes’ alteration status. *EGFR*, *ALK*, *ROS1*, and *RET* mutation status and *EGFR*, *ALK*, *ROS1*, *RET*, *NTRK*, and *MET* mutation status were defined as mutant if an alteration occurred in at least 1 of the genes, independent of other alterations. CN, copy number; MUT, mutant.

Oncogenic driver alterations were identified in baseline ctDNA from 182 patients (36.6% of the BEP); *KRAS* alterations were the most common, found in 20% of patients (Fig. 4A). The median OS was longer with SG versus docetaxel in the WT *KRAS* subgroup (12.5 vs. 10.6 months; HR = 0.75; 95% CI, 0.57–0.98) than in the mutant *KRAS* subgroup (10 vs. 9.3 months; HR = 0.96; 95% CI, 0.59–1.58; Fig. 4B). *KRAS* alterations remained a negative prognostic factor after adjusting for other clinical factors including demographic characteristics and stratification factors. Small numbers of oncogenic driver alterations in *ALK*, *EGFR*, *RET*, and *ROS1* detected in ctDNA limited OS interpretations in this subgroup (Fig. 4C). The larger dataset, combining alterations from baseline ctDNA and eCRFs, showed a numerical, albeit nonsignificant OS benefit with SG versus docetaxel for patients with mutant tumors (median OS, 12.3 vs. 7.9 months; HR = 0.49; 95% CI, 0.24–1.03) and similar OS in patients with WT tumors (median OS, 10.8 vs. 10.1 months; HR = 0.90; 95% CI, 0.72–1.12; Fig. 4D).

**Figure 5.**
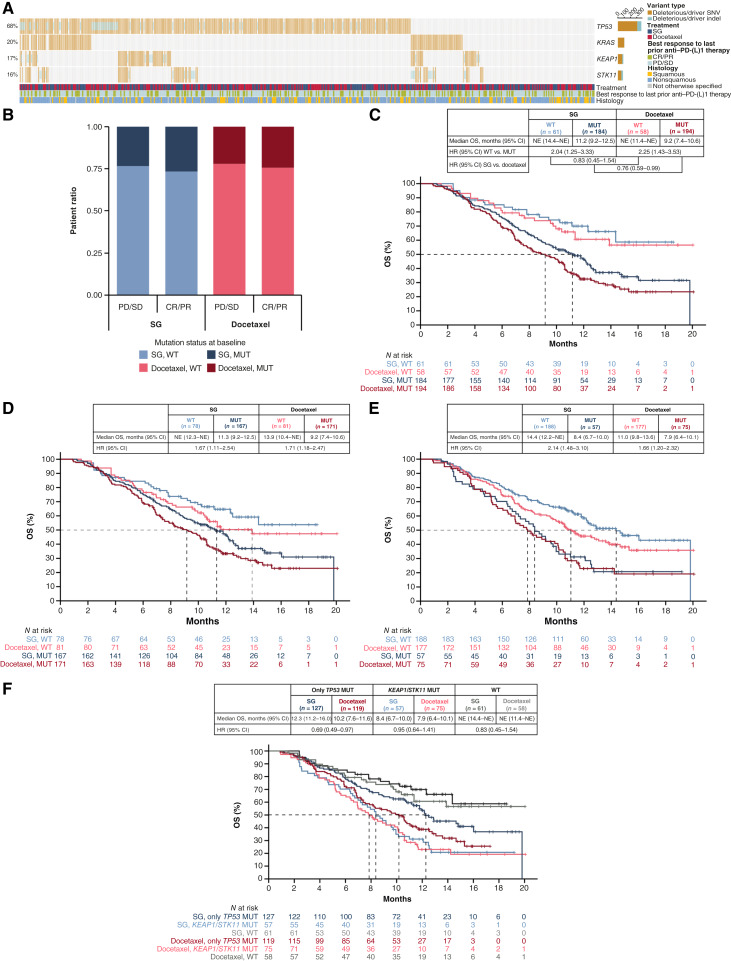
Potential resistance mutations. **A,***TP53*, *STK11*, and *KEAP1* mutation and *KRAS* driver alteration landscape. **B,***TP53*, *STK11*, and *KEAP1* mutation status relative frequency by best response to last prior anti–PD-(L)1–containing therapy and treatment arm. Kaplan–Meier plots for OS by treatment arm and (**C**) *TP53*, *KEAP1*, and/or *STK11* mutation status, (**D**) *TP53* mutation status (independent of other alterations), (**E**) *KEAP1*/*STK11* mutation status (independent of other alterations), and (**F**) *TP53* and *KEAP1/STK11* combined mutation status vs. *TP53* alone vs. no mutations in either of the 3 genes. *TP53*, *KEAP1*, and/or *STK11* mutation status was defined as mutant if mutated in at least 1 of the 3 genes, independent of other genes’ alteration status. *TP53* mutation status was defined as either WT or mutated, independent of other genes’ alteration status. *KEAP1/STK11* mutation status was defined as WT in both genes or mutated in either/both genes, independent of *TP53* or other genes’ alteration status. Only *TP53* mutant (MUT) was defined as *TP53* mutated and both *KEAP1* and *STK11* WT. *KEAP1*/*STK11* MUT was defined as mutated in either/both, irrespective of *TP53* status.

Deleterious mutations in *TP53*, *KEAP1*, and *STK11* were frequent in the ctDNA BEP (Fig. 5A). Most patients (378; 76.1% of the BEP) had at least 1 mutation among *TP53*, *KEAP1*, or *STK11*. *TP53* mutations occurred in 338 of 497 patients (68% of the BEP), of which 246 patients (49% of the BEP) had *TP53* mutations without mutations in *KEAP1* or *STK11*. A total of 40 patients (8% of the BEP) had mutations in *KEAP1* and/or *STK11* but no *TP53* mutations. Irrespective of the treatment arm, the ratio of patients with *TP53*/*KEAP1*/*STK11* (mutated in at least 1 of these genes) mutations was similar between those whose BOR to their last prior PD-(L)1 therapy was CR/PR and those with PD/SD (Fig. 5B).

In both treatment arms, the median OS was longer with WT versus mutant for the *TP53*/*KEAP1*/*STK11* (Fig. 5C), *TP53* (independent of mutation status of other genes; Fig. 5D), and *KEAP1*/*STK11* (mutated in either gene, regardless of *TP53* mutation status; Fig. 5E) groups. An OS benefit was seen with SG versus docetaxel in the mutant *TP53*/*KEAP1*/*STK11* subgroup (median, 11.2 vs. 9.2 months; HR = 0.76; 95% CI, 0.59–0.99; Fig. 5C). Patients harboring tumors with WT *TP53*, *KEAP1*, and *STK11* had the longest survival; the median OS was NE with both SG and docetaxel (HR = 0.83; 95% CI, 0.45–1.54; Fig. 5F). Furthermore, the median OS favored SG over docetaxel in patients with *TP53* mutant and WT *KEAP1*/*STK11* (12.3 vs. 10.2 months; HR = 0.69; 95% CI, 0.49–0.97; Fig. 5F).

**Figure 6.**
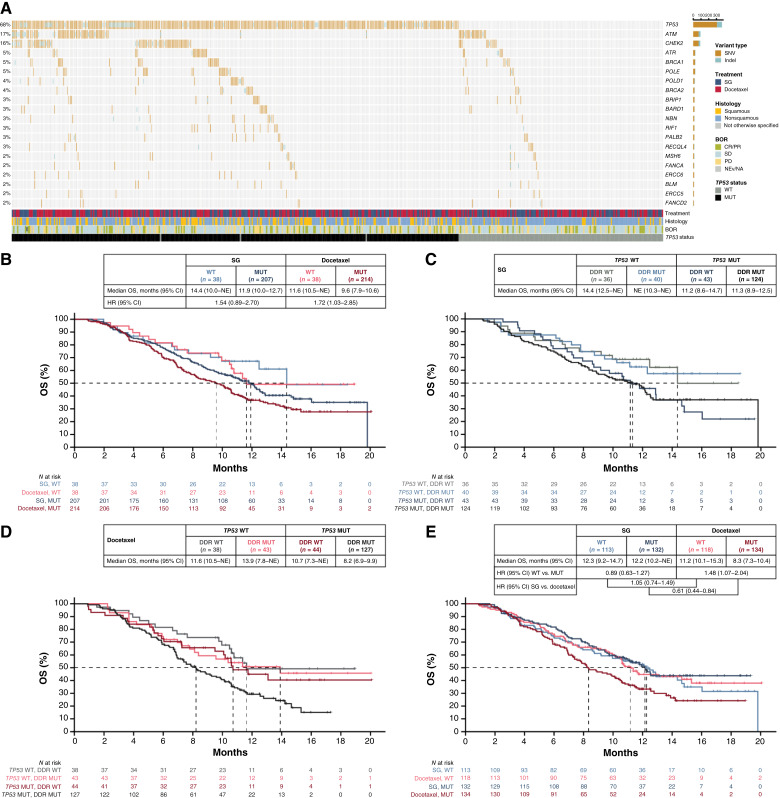
DDR mutations. **A,** Mutation landscape of the 20 most frequently mutated DDR genes in baseline ctDNA. Kaplan–Meier plots for OS by (**B**) KEGG DDR mutation status and treatment arm, (**C**) KEGG DDR and *TP53* mutation status with SG, (**D**) KEGG DDR and *TP53* mutation status with docetaxel, and (**E**) HRR mutation status and treatment arm. MUT, mutant; NA, not assessable; NEv, not evaluable.

To explore potential biomarkers associated with SN-38, KEGG DDR pathway genes were analyzed. At least 1 deleterious mutation was detected in the baseline ctDNA of 421 patients (84.7% of the BEP), affecting 67 of the 80 evaluable KEGG DDR genes (Fig. 6A); *TP53* mutations were the most frequent. Excluding *TP53*, 334 patients (67.2% of the BEP; 164 with SG and 170 with docetaxel) had at least 1 mutation of the remaining 66 DDR genes. The DDR mutation frequency was 81.6% in nonsquamous (Supplementary Fig. S6A) and 92% in squamous histologies (Supplementary Fig. S6B), independent of *TP53* mutation status. The mutation frequency for each gene was similar in both histologies, except *TP53*, for which mutations were more frequent in squamous histology.

With *TP53* mutations present in 68% of patients, the WT subgroups were small, potentially confounding the OS analysis of other DDR mutations (Fig. 6B). *TP53* and other DDR mutations were significantly associated (*χ*^2^ test, *P* < 0.05). After adjusting for *TP53* mutations using a multivariate Cox model, DDR mutations were not a significant biomarker for SG and were a negative predictive factor for docetaxel. *TP53* mutation was associated with worse OS outcomes with SG regardless of DDR mutation status (Fig. 6C). Patients with tumors with *TP53* and DDR co-mutations treated with docetaxel had the shortest median OS (Fig. 6D).

In addition to the KEGG DDR pathways, the HRR mutation subset was explored in the same dataset. Deleterious mutations in HRR occurred in 266 of 497 patients (53.5% of the BEP; 132 with SG and 134 with docetaxel), and each of the 24 genes had at least 1 deleterious mutation (Supplementary Fig. S7). A numerical OS benefit was observed in the HRR mutant subgroup with SG versus docetaxel (median, 12.2 vs. 8.3 months; HR = 0.61; 95% CI, 0.44–0.84; Fig. 6E). These data were consistent when including *TP53* in the multivariate analysis of the HRR subset of genes (Supplementary Fig. S8).

*TOP1* amplifications were observed in 40 of 497 baseline ctDNA samples (8% of the BEP; 18 with SG and 22 with docetaxel). Patients harboring tumors with *TOP1* amplifications had numerically worse OS than those in the WT group, in both the SG (median, 6.7 vs. 12.3 months; HR = 1.28; 95% CI, 0.67–2.43) and docetaxel (median, 6.4 vs. 10.6 months; HR = 2.26; 95% CI, 1.39–3.67) arms, albeit results were limited by the small sample sizes of the *TOP1* amplification subgroups. At baseline, 4 somatic mutations were identified in *TACSTD2* (the gene encoding Trop-2) in 4 patients (2 in each arm), and no *TACSTD2* amplification was detected. *TACSTD2* mutations were not assessed at other timepoints.

## Discussion

In this exploratory analysis from the EVOKE-01 study, we evaluated the protein expression of Trop-2, the binding antigen of SG, as a predictive biomarker and assessed baseline ctDNA levels and ctDNA reduction as indicators of NSCLC disease burden and treatment activity. In addition, we explored biomarkers commonly used in NSCLC treatment decisions or linked to prognosis, including oncogenic driver alterations and mutations involved in therapeutic resistance. Finally, we examined biomarkers linked to sensitivity to the ADC payload SN-38, such as *TOP1* (encoding the SN-38 target TOP1 protein) and other DDR genes.

Studies have shown that SG delivers markedly higher SN-38 levels to tumors, with a better therapeutic index than irinotecan (28–30). SG contains a pH-sensitive, hydrolyzable linker that facilitates rapid internalization and efficient release of SN-38 in Trop-2–expressing cancer cells and into the acidic tumor microenvironment. Following internalization, SN-38 induces DNA damage and results in cell death. SN-38 released into the tumor microenvironment diffuses into neighboring cells in an antigen-independent manner, killing tumor cells irrespective of Trop-2 expression (31). These mechanisms of action enable SG to eliminate cancer cells regardless of Trop-2 expression, supporting broad clinical activity.

In EVOKE-01, Trop-2 expression did not identify patients who benefited more from SG versus docetaxel. These results are consistent with analyses performed in clinical studies of triple-negative and HR+/HER2− breast (32, 33) and urothelial cancers (34, 35), in which Trop-2 expression also did not predict improved outcomes with SG over the control arm or identified a subpopulation of patients who benefited more with SG. Furthermore, in the current study, Trop-2 expression was a negative prognostic factor, with higher Trop-2 expression associated with shorter median OS and PFS. In NSCLC, Trop-2 may be a negative prognostic factor (8–10) or may lack prognostic significance (4). Most published datasets were smaller than EVOKE-01, a global, randomized controlled trial with a Trop-2–directed ADC and a control arm, providing a more rigorous evaluation of Trop-2’s prognostic value in patients with NSCLC receiving second-line or later therapy. These results, although negative, are clinically informative as they temper expectations for Trop-2 as a predictive biomarker for populations with NSCLC receiving Trop-2–directed ADCs, enabling researchers to refine testing methods beyond traditional IHC.

Although SG and datopotamab deruxtecan are both anti–Trop-2 ADCs, substantial differences in linker chemistry, payload design, dependence on Trop-2 membrane expression, and internalization efficiency exist. The hydrolyzable linker design of SG permits extracellular release of SN-38, potentially enhancing the bystander effect and may be related to the activity of SG in both squamous and nonsquamous NSCLC histologies. Datopotamab deruxtecan relies on an enzymatically cleavable linker, and recent evidence suggests that Trop-2 membrane expression and efficient internalization are required for optimal activity. For this reason, quantitative pathology approaches such as quantitative continuous scoring normalized membrane ratio (QCS-NMR TROP-2 QCS-NMR) were proposed to measure the internalization capacity (36). In contrast to datopotamab deruxtecan, SG efficacy did not depend on Trop-2 expression (36). Consistent with this distinction, results from the phase II OptiTROP-Lung01 study evaluating sacituzumab tirumotecan plus the anti–PD-L1 antibody tagitanlimab indicated that manual Trop-2 assessment did not correlate with clinical benefit in patients treated with sacituzumab tirumotecan (37), supporting the hypothesis that ADC design may influence the predictive relevance of Trop-2 expression. Bessede and colleagues (11) suggested that Trop-2 expression may correlate with resistance to immune checkpoint inhibition in patients with advanced NSCLC. In EVOKE-01, patients were not treated with immunotherapy, but they were stratified by best response to last anti–PD-(L)1–containing regimen. In the ITT population, efficacy differed between patients who achieved PD/SD and those with CR/PR (5). Our analysis revealed no difference in Trop-2 expression between these subgroups that could explain the data observed in the ITT population.

High baseline ctDNA was a negative prognostic factor and was associated with shorter median OS and PFS with both SG and docetaxel. Moreover, ctDNA reduction was observed in both treatment arms, supporting SG as an active therapy in metastatic NSCLC. Our observations align with previous studies showing that ctDNA reduction following treatment initiation predicts better clinical outcomes, whereas persistent or increasing ctDNA levels signal poor response or impending relapse (16–18, 38). Although these findings are not unique to this dataset, these results are clinically informative, as they include samples from a randomized controlled trial as opposed to an observational study, aligning with calls for further evaluation of ctDNA as a prognostic biomarker in this setting. Additionally, although heterogeneity in ctDNA methodologies across studies may limit direct comparisons, these findings utilized a reproducible assay approach, adding to the growing body of evidence and contributing toward standardization of methods and subsequent clinical implementation of ctDNA analyses (39).

EVOKE-01 required prior targeted therapy for patients with known AGAs, resulting in fewer patients with driver alterations compared with general NSCLC populations. *KRAS* mutations were the predominant oncogenic driver alteration, consistent with previous findings (40, 41), and their role as a negative prognostic factor (21) was reaffirmed. Although a numerical OS improvement was noted with SG versus docetaxel among patients with oncogenic driver alterations in *ALK*, *EGFR*, *RET*, and *ROS1*, the small sample size in the study limits definitive conclusions. Similarly, the TROPION-Lung01 trial demonstrated a numerical OS improvement with a different Trop-2 ADC, datopotamab deruxtecan, versus docetaxel in patients with nonsquamous NSCLC with AGAs (42).

The cytotoxic payload of Trop-2–directed ADCs such as SG induces DNA damage, which in addition to direct tumor cell killing may include an immune-mediated component through the induction of immunogenic cell death, defined as a regulated form of cell death that promotes T cell–mediated immune responses (43). Genomic alterations such as *STK11* and *KEAP1* are associated with immune-suppressed tumor microenvironments (44, 45), whereas *TP53* alterations have been linked with changes in tumor immunogenicity in NSCLC (46). Therefore, the efficacy of SG in relation to the immune-modifying effects of genomic alterations, such as *TP53*, *STK11*, and *KEAP1*, is of interest.

Mutations in *TP53* and *KEAP1/STK11*, previously described as contributing to resistance to PD-(L)1 therapy, were identified as negative prognostic factors, either individually or in combination (21, 23). Although an OS benefit was observed with SG versus docetaxel in the *TP53*-mutant subgroup, interpretation remains limited because of the small sample size of the WT subgroup, as 68% of patients had *TP53* mutations. In addition, analysis of mutation frequency across the subgroups with best response to last prior PD-(L)1 therapy (CR/PR vs. PD/SD) showed no difference in mutation frequency that would explain the differences in efficacy observed in these subgroups in the ITT population (5).

DDR responses may influence sensitivity to TOP1 inhibitors like SN-38 by resolving SN-38–stabilized cytotoxic TOP1 cleavage complexes (47). DDR genes, including *TOP1* and other DNA repair genes, could affect SN-38 and SG cytotoxicity. Studies report varying DDR mutation frequencies in NSCLC across gene sets (48–50). Here, characterization of the DDR mutation landscape, employing broader (KEGG DDR) and more focused (HRR) gene sets, revealed frequent deleterious mutations. Consistent with the literature, the most frequently mutated DDR genes included *ATM*, *ATR*, and Fanconi anemia genes. In EVOKE-01, DDR deficiency did not predict benefit with SG versus docetaxel; in contrast, an OS benefit was seen in both DDR mutant and WT subgroups with SG. The analysis revealed a potential OS benefit with SG in the HRR mutant subgroup; however, the high prevalence of *TP53* mutations in the patient population confounded this analysis. HRR-deficient tumors are sensitive to DNA-damaging agents such as platinum chemotherapy and poly(ADP-ribose) polymerase inhibitors (51, 52), which may explain the efficacy difference between SG and docetaxel in the HRR mutant subgroup seen in the present study. Consistent with our results, SG provided benefit in metastatic HR+/HER2− breast cancer regardless of DDR mutation status (7). *TOP1* point mutations that may confer SN-38 resistance were infrequent in this study and could not be evaluated. *TOP1* copy-number increase has been associated with high sensitivity to SN-38 (53). Here, we observed shorter OS across treatment arms when *TOP1* was amplified; however, only 40 patients with these tumor alterations were identified, limiting interpretation.

In conclusion, extensive biomarker analyses of samples collected in the EVOKE-1 study did not identify a subpopulation of patients that benefited more from SG versus docetaxel. In particular, neither Trop-2 expression (the biomarker associated with the mechanism of action of SG) nor DDR mutations that may sensitize to SN-38 were predictive for SG benefit. ctDNA reduction was observed in both arms, supporting that SG is an active agent in this setting. High baseline ctDNA levels and a smaller or lack of ctDNA reduction on treatment also correlated with poorer prognosis. In addition, *TP53*, *STK11*, *KEAP1*, and *KRAS* mutations were negative prognostic indicators, with the *TP53*-mutant subgroup experiencing better outcomes with SG than with docetaxel. Due to the study design, data on oncogenic driver alterations (*EGFR* and *ALK*) were limited, and the activity of SG in patients with oncogenic alterations should be explored further. Future research should continue investigating predictive biomarkers for SG response in NSCLC to advance precision medicine approaches and improve patient outcomes.

## Supplementary Material

Supplementary Table S1 Baseline demographics and disease characteristics.

Supplementary Table S2 Representativeness of study patients

Supplementary Table S3 Response rates by treatment arm and Trop-2 median H-score subgroup in the Trop-2 biomarker evaluable population.

Supplementary Figure S1 Kaplan-Meier plots for OS by treatment arm for the (A) ITT, (B) Trop-2 biomarker evaluable population, and (C) ctDNA biomarker evaluable population.

Supplementary Figure S2 Trop-2 subgroup analysis of OS.

Supplementary Figure S3 Kaplan-Meier plots for OS for median Trop-2 H-score subgroups by treatment arm among patients with (A) PD/SD and (B) CR/PR as best response to last prior anti-PD-(L)1–containing therapy.

Supplementary Figure S4 Trop-2 subgroup analysis of PFS.

Supplementary Figure S5 ctDNA level (A) Methylation-based ctDNA fraction at baseline and C2D1 by treatment arm. Kaplan-Meier plots for PFS for baseline ctDNA median subgroups with (B) SG and (C) docetaxel.

Supplementary Figure S6 Mutation landscape of the 20 most frequently mutated DDR genes in baseline ctDNA for patients with (A) nonsquamous and (B) squamous histology, adjusted for TP53.

Supplementary Figure S7 Genomic alteration landscape based on the HRR 24-gene list.

Supplementary Figure S8 DDR pathway forest plot analysis, adjusted for TP53.

## Data Availability

Gilead Sciences, Inc., shares anonymized individual patient data upon request or as required by law or regulation with qualified external researchers based on submitted curriculum vitae and a nonconflict of interest. The request proposal must also include a statistician. Data requests should be sent to datasharing@gilead.com. Approval of such requests is at Gilead Science’s discretion and is dependent on the nature of the request, the merit of the research proposed, the availability of the data, and the intended use of the data.
