# Supplementary material for: Evaluation of Biomarkers for Sacituzumab Govitecan in Metastatic Non–Small Cell Lung Cancer: Insights From the EVOKE-01 Study
Source: Cancer Res Commun. 2026 Aug 6;6(8):1875–88. doi: 10.1158/2767-9764.CRC-26-0070 (PMC13446097; doi:10.1158/2767-9764.CRC-26-0070)
Supplement: Supplementary file 1 [file crc-26-0070_supplementary_table_s1_suppst1.docx]

| **Supplementary Table S1. Baseline Demographic and Disease Characteristics** | | | | | | |
| --- | --- | --- | --- | --- | --- | --- |
| Characteristic | ITT | | Trop-2 Biomarker-Evaluable Set | | ctDNA Biomarker-Evaluable Set | |
|  | SG (n = 299) | Docetaxel  (n = 304) | SG  (n = 193) | Docetaxel  (n = 187) | SG  (n = 245) | Docetaxel  (n = 252) |
| Median age, years (range) | 66 (31-84) | 64 (32-83) | 66 (31-84) | 64 (32-83) | 65 (31-83) | 64 (32-83) |
| < 65 years, n (%) | 136 (45.5) | 161 (53.0) | 83 (43.0) | 102 (54.5) | 114 (46.5) | 135 (53.6) |
| ≥ 65 years, n (%) | 163 (54.5) | 143 (47.0) | 110 (57.0) | 85 (45.5) | 131 (53.5) | 117 (46.4) |
| Male, n (%) | 194 (64.9) | 216 (71.1) | 125 (64.8) | 136 (72.7) | 157 (64.1) | 182 (72.2) |
| Race or ethnic group, n (%) |  |  |  |  |  |  |
| Asian | 17 (5.7) | 26 (8.6) | 13 (6.7) | 21 (11.2) | 15 (6.1) | 25 (9.9) |
| Black | 6 (2.0) | 7 (2.3) | 3 (1.6) | 6 (3.2) | 5 (2.0) | 6 (2.4) |
| White | 229 (76.6) | 216 (71.1) | 147 (76.2) | 124 (66.3) | 188 (76.7) | 172 (68.3) |
| Other/not specified/not reported*^a^* | 47 (15.7) | 55 (18.1) | 30 (15.5) | 36 (19.3) | 37 (15.1) | 49 (19.4) |
| Geographic region, n (%) |  |  |  |  |  |  |
| US | 32 (10.7) | 28 (9.2) | 15 (7.8) | 13 (7.0) | 29 (11.8) | 22 (8.7) |
| Canada/Western EU/Australia | 224 (74.9) | 231 (76.0) | 148 (76.7) | 146 (78.1) | 179 (73.1) | 189 (75.0) |
| Rest of world | 43 (14.4) | 45 (14.8) | 30 (15.5) | 28 (15.0) | 37 (15.1) | 41 (16.3) |
| Histology, n (%) |  |  |  |  |  |  |
| Squamous | 84 (28.1) | 80 (26.3) | 61 (31.6) | 57 (30.5) | 68 (27.8) | 70 (27.8) |
| Nonsquamous | 204 (68.2) | 220 (72.4) | 124 (64.2) | 129 (69.0) | 170 (69.4) | 178 (70.6) |
| Not otherwise specified | 11 (3.7) | 4 (1.3) | 8 (4.1) | 1 (0.5) | 7 (2.9) | 4 (1.6) |
| Best response to prior immunotherapy, n (%) |  |  |  |  |  |  |
| CR/PR | 106 (35.5) | 113 (37.2) | 69 (35.8) | 73 (39.0) | 86 (35.1) | 94 (37.3) |
| PD/SD | 192 (64.2) | 191 (62.8) | 123 (63.7) | 114 (61.0) | 158 (64.5) | 158 (62.7) |
| Missing | 1 (0.3) | 0 | 1 (0.5) | 0 | 1 (0.4) | 0 |
| Received targeted therapy for AGA, n (%) | 19 (6.4) | 25 (8.2) | 13 (6.7) | 21 (11.2) | 11 (4.5) | 21 (8.3) |
| ECOG PS score, n (%) |  |  |  |  |  |  |
| 0 | 101 (33.8) | 89 (29.3) | 62 (32.1) | 62 (33.2) | 85 (34.7) | 75 (29.8) |
| 1 | 198 (66.2) | 212 (69.7) | 131 (67.9) | 124 (66.3) | 160 (65.3) | 175 (69.4) |
| 2 | 0 | 1 (0.3) | 0 | 0 | 0 | 0 |
| Missing | 0 | 2 (0.7) | 0 | 1 (0.5) | 0 | 2 (0.8) |
| Number of prior anticancer therapies, n (%) |  |  |  |  |  |  |
| 1 | 167 (55.9) | 167 (54.9) | 105 (54.4) | 99 (52.9) | 133 (54.3) | 137 (54.4) |
| 2 | 103 (34.4) | 101 (33.2) | 71 (36.8) | 62 (33.2) | 91 (37.1) | 84 (33.3) |
| 3 | 24 (8.0) | 25 (8.2) | 15 (7.8) | 15 (8.0) | 18 (7.3) | 20 (7.9) |
| 4 | 3 (1.0) | 9 (3.0) | 2 (1.0) | 9 (4.8) | 2 (0.8) | 9 (3.6) |
| 5 | 2 (0.7) | 2 (0.7) | 0 | 2 (1.1) | 1 (0.4) | 2 (0.8) |

*^a^*Other includes American Indian or Alaska Native, Native Hawaiian or other Pacific Islander, or other.
AGA, actionable genomic alteration; CR, complete response; ctDNA, circulating tumor DNA; ECOG PS, Eastern Cooperative Oncology Group performance status; EU, European Union; ITT, intent-to-treat; PD, progressive disease; PR, partial response; SD, stable disease; SG, sacituzumab govitecan; Trop-2, trophoblast cell surface antigen 2; US, United States.
