# Supplementary material for: Evaluation of Biomarkers for Sacituzumab Govitecan in Metastatic Non–Small Cell Lung Cancer: Insights From the EVOKE-01 Study
Source: Cancer Res Commun. 2026 Aug 6;6(8):1875–88. doi: 10.1158/2767-9764.CRC-26-0070 (PMC13446097; doi:10.1158/2767-9764.CRC-26-0070)
Supplement: Supplementary file 2 [file crc-26-0070_supplementary_table_s2_suppst2.docx]

**Supplementary Table S2.** Representativeness of study patients

| **Category** | **Description** |
| --- | --- |
| Cancer type(s)/subtype(s)/ stage(s)/condition | Metastatic NSCLC |
| **Conditions related to:** | |
| Sex | Globally, 2.5 million new cases of lung cancer were estimated in 2022, with NSCLC comprising the majority (80–85%) of these cases (1). Records from the SEER-18 database from the US from 2010 to 2017 show more patients were men (53%) (2).  Although NSCLC has a higher overall incidence in men, women ≤49 years have a higher probability of developing lung cancer than men (1 in 783 vs 1 in 901) (3,4). |
| Age | The median age of diagnosis of NSCLC is 70 years (2), with 68% and 30–40% of cases diagnosed in people aged ≥65 years and ≥75 years, respectively (2,5).  Younger adults are more likely to be diagnosed with stage IV disease, while a higher proportion of older patients were diagnosed with stage I disease (2). |
| Race/ethnicity | According to data from the SEER-18 database (2007–2018), the majority of patients with NSCLC from the US are White (75%), 12% Black, 6% Asian or Pacific Islander, 6% Hispanic, and <1% are American Indian or Alaskan Native (6).  The incidence rates of NSCLC vary according to race and ethnicity, with the highest incidence across all stages observed in Black men, followed by White men. The lowest incidence of NSCLC was observed in Hispanic people (6). |
| Geography | An estimated 226,650 new cases of lung and bronchus cancer are expected in the US during 2025 (3). Geographically, lung cancer mortality may be higher in rural areas due to lower screening rates from a lack of access and higher smoking rates in rural populations (7). Greater 5-year survival rates are associated with higher socioeconomic status, where individuals from urban areas are more likely to have a higher socioeconomic status (7). |
| Other considerations | The inclusion of only patients with stable, previously treated brain metastases (among those with such metastases) may underrepresent real-world NSCLC populations, which may include patients with active or untreated brain metastases. AGAs are present in approximately 25% of NSCLC tumors (1), whereas in the EVOKE-01 study, 7% of patients with NSCLC had an AGA. Patients with NSCLC positive for known AGAs were required to have received prior treatment with ≥1 tyrosine kinase inhibitor and have disease that had progressed after platinum-based chemotherapy in combination or sequential with an anti–PD-(L)-1–containing regimen. Targeted therapy is the standard of care in NSCLC positive for AGAs, with platinum-based chemotherapy possibly considered as subsequent therapy (8). Consequently, based on the requirement for patients with AGAs to have received all 3 regimens, the study population may not be representative of the broader population. In addition, the study enrolled previously treated patients with metastatic NSCLC, whereas real-world patients would have differing levels of disease burden. |
| Overall representativeness in this study | The present manuscript is an exploratory analysis from the phase III EVOKE-01 study seeking to identify predictive biomarkers for sacituzumab govitecan. Patient demographic and baseline characteristics data for EVOKE-01 were reported previously (9). This study evaluated a total of 603 patients with previously treated nonsquamous or squamous metastatic NSCLC and included patients from sites in 20 countries across North and South America, Europe, Asia, and Australia. The study demographics, including median age (65 years), sex (68% men), race, and ethnicity of this study sample broadly mirrored that of the NSCLC population in the US according to SEER-18 data (53% men; median age at diagnosis of 70 years) (2); differences in patient representativeness compared with the literature may be due to small sample size and that patients in this study had advanced disease. |
| Abbreviations: AGA, actionable genomic alteration; NSCLC, non–small cell lung cancer; PD-(L)-1, programmed cell death protein (ligand) 1; SEER, Surveillance, Epidemiology, and End Results; US, United States.  References:   1. Li T, Ma W, Al-Obeidi E. Evolving precision first-line systemic treatment for patients with unresectable non-small cell lung cancer. *Cancers (Basel*). 2024; 26;16:2350. 2. Ganti AK, Kelin AB, Cotarla I, Seal B, Chou E. Update of incidence, prevalence, survival, and initial treatment in patients with non-small cell lung cancer in the US. *JAMA Oncol.* 2021;7:1824–32. 3. Siegel RL, Kratzer TB, Giaquinto AN, Sung H, Jemal A. Cancer statistics, 2025. *CA Cancer J Clin*. 2025;75:10–45. 4. Jemal A, Schafer EJ, Sung H, Bandi P, Kratzer T, Islami F, et al*.* The burden of lung cancer in women compared with men in the US. *JAMA Oncol.* 2023;9:1727–8. 5. Aimalla N, Abughanimeh O. Survival and incidence trends of non-small cell lung cancer in elderly patients (>75 years): insights from SEER database. *J Thor Oncol.* 2024;19(Suppl):S587–8. 2024 World Conference on Lung Cancer abstract EP.08F. 6. Primm KM, Zhao H, Hernandez DC, Chang S. Racial and ethnic trends and disparities in NSCLC. *JTO Clin Res Rep.* 2022;3:100374. 7. Kurzrock R, Chaudhuri AA, Feller-Kopman D, Florez N, Gorden J, Wistuba II. Healthcare disparities, screening, and molecular testing in the changing landscape of non-small cell lung cancer in the United States: a review. *Cancer Metastasis Rev*. 2024;43:1217–31. 8. Yamaguchi T, Shimizu J, Matsuzawa R, Watanabe N, Horio Y, and Fujiwara Y*.* Efficacy of chemotherapy plus immune checkpoint inhibitors in patients with non-small cell lung cancer who have rare oncogenic driver mutations: a retrospective analysis. *BMC Cancer.* 2024;24:842. 9. Paz-Ares LG, Juan-Vidal O, Mountzios GS, Felip E, Reinmuth N, de Marinis F, et al. Sacituzumab govitecan versus docetaxel for previously treated advanced or metastatic non-small cell lung cancer: the randomized, open-label phase III EVOKE-01 study. *J Clin Oncol*. 2024;42:2860–72. | |
