# Supplementary material for: Evaluation of Biomarkers for Sacituzumab Govitecan in Metastatic Non–Small Cell Lung Cancer: Insights From the EVOKE-01 Study
Source: Cancer Res Commun. 2026 Aug 6;6(8):1875–88. doi: 10.1158/2767-9764.CRC-26-0070 (PMC13446097; doi:10.1158/2767-9764.CRC-26-0070)
Supplement: Supplementary file 3 [file crc-26-0070_supplementary_table_s3_suppst3.docx]

| **Supplementary Table S3. Response Rates by Treatment Arm and Trop-2 Median H-score Subgroup in the Trop-2 BEP** | | | |
| --- | --- | --- | --- |
| **Subgroup** | **Patients, n** | **ORR (95% CI), %** | **DCR (95% CI), %** |
| **H-score < 190** |  |  |  |
| SG | 94 | 14.9 (8.4-23.7) | 67.0 (56.6-76.4) |
| Docetaxel | 92 | 20.7 (12.9-30.4) | 71.7 (61.4-80.6) |
| **H-score ≥ 190** |  |  |  |
| SG | 99 | 11.1 (5.7-19.0) | 68.7 (58.6-77.6) |
| Docetaxel | 95 | 16.8 (9.9-25.9) | 68.4 (58.1-77.6) |

BEP, biomarker evaluable population; CI, confidence interval; DCR, disease control rate; H-score, histological score; ORR, objective response rate; SG, sacituzumab govitecan; Trop-2, trophoblast cell surface antigen 2.
